# Sphingosine 1-phosphate receptor 3 and RhoA signaling mediate inflammatory gene expression in astrocytes

**DOI:** 10.1186/s12974-017-0882-x

**Published:** 2017-06-02

**Authors:** Stephanie S. Dusaban, Jerold Chun, Hugh Rosen, Nicole H. Purcell, Joan Heller Brown

**Affiliations:** 1 Department of Pharmacology, School of Medicine, University of California San Diego, 9500 Gilman Drive, Biomedical Sciences Building Room 3024, La Jolla, CA 92093-0636 USA; 2 Sanford Burnham Prebys Medical Discovery Institute, La Jolla, CA 92037 USA; 3 Department of Chemical Physiology, The Scripps Research Institute, La Jolla, CA 92037 USA

**Keywords:** Astrocytes, Central nervous system, Inflammation, RhoA, S1P, S1P_3_

## Abstract

**Background:**

Sphingosine 1-phosphate (S1P) signals through G protein-coupled receptors to elicit a wide range of cellular responses. In CNS injury and disease, the blood-brain barrier is compromised, causing leakage of S1P from blood into the brain. S1P can also be locally generated through the enzyme sphingosine kinase-1 (Sphk1). Our previous studies demonstrated that S1P activates inflammation in murine astrocytes. The S1P_1_ receptor subtype has been most associated with CNS disease, particularly multiple sclerosis. S1P_3_ is most highly expressed and upregulated on astrocytes, however, thus we explored the involvement of this receptor in inflammatory astrocytic responses.

**Methods:**

Astrocytes isolated from wild-type (WT) or S1P_3_ knockout (KO) mice were treated with S1P_3_ selective drugs or transfected with short interfering RNA to determine which receptor subtypes mediate S1P-stimulated inflammatory responses. Interleukin-6 (IL-6), and vascular endothelial growth factor A (VEGFa) messenger RNA (mRNA) and cyclooxygenase-2 (COX-2) mRNA and protein were assessed by q-PCR and Western blotting. Activation of RhoA was measured using SRE.L luciferase and RhoA implicated in S1P signaling by knockdown of Gα_12/13_ proteins or by inhibiting RhoA activation with C3 exoenzyme*.* Inflammation was simulated by in vitro scratch injury of cultured astrocytes.

**Results:**

S1P_3_ was highly expressed in astrocytes and further upregulated in response to simulated inflammation. Studies using S1P_3_ knockdown and S1P_3_ KO astrocytes demonstrated that S1P_3_ mediates activation of RhoA and induction of COX-2, IL-6, and VEGFa mRNA, with some contribution from S1P_2_. S1P induces expression of all of these genes through coupling to the Gα_12/13_ proteins which activate RhoA. Studies using S1P_3_ selective agonists/antagonists as well as Fingolimod (FTY720) confirmed that stimulation of S1P_3_ induces COX-2 expression in astrocytes. Simulated inflammation increased expression of Sphk1 and consequently activated S1P_3_, demonstrating an autocrine pathway through which S1P is formed and released from astrocytes to regulate COX-2 expression.

**Conclusions:**

S1P_3_, through its ability to activate RhoA and its upregulation in astrocytes, plays a unique role in inducing inflammatory responses and should be considered as a potentially important therapeutic target for CNS disease progression.

## Background

Sphingosine 1-phosphate (S1P) is a bioactive lipid and G protein-coupled receptor (GPCR) ligand formed within the brain from sphingomyelin and is also present at high levels in blood where it is bound to lipoproteins and stored in erythrocytes [1–5]. There are five S1P receptor subtypes [6, 7] with S1P_1_, S1P_2_, S1P_3_, and S1P_5_ (and in some reports S1P_4_) expressed in the CNS [8–13]. Astrocytes are activated in response to CNS injury and diseases like multiple sclerosis (MS) and undergo astrogliosis characterized by increases in proliferation, hypertrophy, and glial fibrillary acidic protein (GFAP) expression [14–20]. S1P induces astrogliosis when injected into the brain as evidenced by increases in GFAP expression and astrocyte proliferation [21–23]. The importance of S1P receptors in disease is highlighted by the widespread acceptance of Fingolimod (FTY720; Gilenya) as a first line oral drug to treat MS [24–27]. Phosphorylated fingolimod functions as an S1P analogue that blocks lymphocyte egress through functional inhibition of S1P_1_ signaling [28, 29]. Its efficacy in the EAE mouse model of MS has also been linked to signaling through S1P_1_ on astrocytes [30].

The predominant S1P receptor subtype detected by quantitative-PCR (q-PCR) in cortical astrocytes is S1P_3_, although S1P_1_ is also expressed on astrocytes from rat and mouse brain [8, 12, 31]. The potential importance of S1P_3_ signaling in astrocytes is suggested by the finding that this receptor is upregulated in MS lesions and in response to inflammatory stimuli [32–34]. In a mouse model of Sandhoff disease characterized by neuronal death and astrocyte proliferation, deletion of S1P_3_, along with the enzyme sphingosine kinase (Sphk) which catalyzes the synthesis of S1P, decreased astrogliosis and disease severity [35]. Importantly, whereas S1P_1_ exclusively couples to the G protein Gα_i_, S1P_3_ couples promiscuously and its coupling to Gα_12/13_ activates the small G-protein RhoA [36–38]. Previous work from our laboratory documented the importance of RhoA activation in inducing astrocyte proliferation, gene expression, and inflammation in response to stimulation of GPCRs for thrombin and S1P [39–44].

Here, we ask whether stimulation of the S1P_3_ receptor on astrocytes activates RhoA, is responsible for inflammatory gene expression, or can be locally engaged by endogenously formed S1P in an in vitro model of neuroinflammation. We demonstrate that S1P_3_, and not S1P_1_, mediates induction of interleukin-6 (IL-6) and vascular endothelial growth factor A (VEGFa) mRNA, and cyclooxygenase-2 (COX-2) mRNA and protein in mouse astrocytes and that this occurs through S1P receptor coupling to Gα_12/13_ and RhoA. We also demonstrate that simulated inflammation in vitro leads to increases in expression of Sphk1 and S1P_3_ which could contribute to autocrine inflammatory astrocyte signaling.

## Methods

### Agonists and inhibitors

Sources were as follows: S1P was obtained from Avanti Polar Lipids. The S1P_3_ antagonist SPM-354 was synthesized and characterized as described [45]. A cell permeable botulinum C3 toxin exoenzyme, which inhibits RhoA activation, was obtained from cytoskeleton. Pertussis toxin, which ribosylates and inactivates the alpha subunit of the G_i_ protein, was used to block signaling through receptor coupling to G_i_ (Tocris Bioscience). The S1P_3_ specific agonist CYM-51736 was provided by the Rosen laboratory, and the functional S1P_1_ antagonist and MS therapeutic, FTY720 (Fingolimod), *and S1P*_*2*_ *antagonist JTE-013* was from Cayman Chemicals. S1P was used at 0.5 μM. SPM-354 was used at 5 μM. C3 exoenzyme was used at 0.5 μg/mL. FTY720 was used at 100 nM. CYM-51736 was used at 10 μM, *JTE-013 at 1* μM, and pertussis toxin at 100 ng/ml.

### Animals

All procedures were performed in accordance with NIH Guide and Care and Use of Laboratory Animals and approved by the Institutional Animal Care and Use Committee at the University of California San Diego. C57BL/6 wild-type and homozygous S1P_3_ KO mice which do not exhibit any gross phenotypic abnormalities [36, 46] were used for astrocyte isolation.

### Primary culture of astrocytes

Astrocytes were isolated from P1-P3 postnatal WT and S1P_3_ KO mice [44]. Purity of astrocytes was determined to be ~95% based on GFAP staining. In all experiments, WT and S1P_3_ KO astrocytes were used at passage 2. Astrocytes were cultured in six-well plates, maintained in high-glucose DMEM supplemented with 10% FBS/2 mM glutamine/100 units/ml penicillin/100 μg/ml streptomycin (Invitrogen, Carlsbad, CA) at 37 °C in a humidified 5% CO_2_-incubator. Cells used for experiments were at 80% confluency and serum-starved for 18–24 h prior to agonist treatment.

### siRNA transfections

Pre-designed mouse short interfering RNA (siRNA) smartpools for S1P_1_, S1P_2_, S1P_3_, and control siRNA were purchased from Bioneer. Pre-designed mouse siRNA for Gα_12_, Gα_13_, Gα_q_, and sphingosine kinase 1 were purchased from Qiagen, individual siRNAs were tested for knockdown, and the most efficient was selected for use in the current studies. Control siRNA was compared to targeted siRNAs used throughout. WT astrocytes on six-well plates were transfected using DharmaFECT-3 transfection reagent (Thermo Scientific) and 2 μM siRNA in a 1:3 ratio respectively. Reagent and siRNA were incubated alone in OPTI-MEM media (Gibco) at room temperature for 10 min followed by mixing and incubating further for 20 min. The siRNA/DharmaFECT-3 mixture was added to plates containing fresh media. Following overnight incubation, media containing siRNA was removed, and cells were washed. Astrocytes were serum-starved for 18–24 h prior to treatment. The table below lists the predesigned or three smartpool siRNA sequences used in these studies.

**Table:** 

*siRNA*	*Sense Sequence*	*Antisense Sequence*
S1P_1_	GAUAUCAUAGUCCGGCAUU CCGGAGCUUUGAUUUUGCA CGGACCUAUUAGCAGGCGU	AAUGCCGGACUAUGAUAUC UGCAAAAUCAAAGCUCCGG ACGCCUGCUAAUAGGUCCG
S1P_2_	CUGUACGUCCGAAUCUACU CACUUCUGGAGUGCCAGUA CCUCGGUCUUUAGCCUCCU	AGUAGAUUCGGACGUACAG UACUGGCACUCCAGAAGUG AGGAGGCUAAAGACCGAGG
S1P_3_	UCUUGGUCACCUGUAGCUU UGUACAGGAUGUAUACGAU AGACAUCGGGUGCAUCCAA	AAGCUACAGGUGACCAAGA AUCGUAUACAUCCUGUACA UUGGAUGCACCCGAUGUCU
Gα_12_	UGACUUCGUUAUAAAGAAATT	UUUCUUUAUAACGAAGUCATG
Gα_13_	CCAUAAUCCUCUUCUUAAATT	UUUAAGAAGAGGAUUAUGGAG
Gα_q_	GGUGGAUAGUAUUAUCCUATT	UAGGAUAAUACUAUCCACCAG
Sphingosine kinase 1	CGAGCAGGUGACUAAUGAATT	UUCAUUAGUCACCUGCUCGTA

### SRE.L luciferase assay

Astrocytes were cultured on 12-well plates and transfected with 500 ng of SRE.L and 50 ng of Renilla as an internal control using DharmaFECT-3 as described above. Following overnight incubation, cells were serum-starved for 18–24 h prior to S1P treatment for 8 h. Cells were lysed, and luciferase activities were measured using the Dual-Luciferase Reporter Assay System (Promega).

### In vitro scratch *injury* model

WT or S1P_3_ KO astrocytes were cultured on six-well plates and grown to confluence followed by serum starvation for 18–24 h. To stimulate astrogliosis and inflammation, plates were scratched with a 200-μL pipette tip six times (three vertical and three horizontal) across the dish [47]. Cells were harvested and lysed after 1 h scratch for mRNA analysis or after 8 h for Western blotting as described below.

### q-PCR

For gene expression analyses, RNA was extracted from astrocytes using an RNeasy kit (Invitrogen) [40]. Complementary DNA (cDNA) was synthesized with High-Capacity cDNA Reverse Transcription Kit (Applied Biosystems ABI) and real-time q-PCR performed with TaqMan Universal Master Mix II, with UNG (Applied Biosystems ABI). To analyze gene expression in mouse astrocytes treated with S1P or scratch wounding, gene-specific primers for COX-2, IL-6, VEGFa, S1P_1_, S1P_2_, S1P_3_, Sphk1, and GAPDH (as an internal control) were used (Integrated DNA Technologies). S1P_3_ KO astrocytes were analyzed for the levels of S1P_1_ and S1P_2_ and were found to have no significant compensatory changes (data not shown). Data were normalized to internal GAPDH, and fold change was determined according to a published protocol [48]. Values for comparison for a single gene across multiple samples was determined using cycle threshold (Ct) data fitted to a standard curve. For comparison of multiple transcripts in a single sample, then the 2^−ΔΔCt^ method was applied to the Ct value [48].

### Western blotting

Astrocytes were lysed in RIPA buffer (20 mm Tris, 250 mm NaCl, 3 mm EDTA, 3 mm EGTA, and 20 mm β-glycerophosphate) supplemented with sodium vanadate, leupeptin, aprotinin, *p*-nitrophenyl phosphate, and phenylmethylsulfonyl fluoride. BCA analysis was performed using the Micro BCA Protein Assay Kit (ThermoFisher Scientific) to determine protein concentration. Equal amounts of protein (10 μg) were loaded onto 4–12% 10-well or 15-well SDS-PAGE gels (Invitrogen NuPage System). Gels were transferred to PVDF membranes (Millipore), and the resulting blot was probed with specific antibodies. The COX-2 antibody (Cayman #160126) was used at 1:500 dilution, and the band running at 72 kDa band was quantitated. The GAPDH antibody (Cell Signaling Technology #2118) was used at 1:1000 dilution, and a band at 37 kDa was quantitated. Rabbit secondary antibody was used at 1:4000 dilution. Fold changes were determined by densitometry and normalized to accompanying GAPDH blots.

### Statistical analysis

Statistical differences were determined using Tukey’s multicomparison analysis following one-way ANOVA with Prism software (GraphPad). *p* < 0.05 was considered significant.

## Results

### S1P_3_ is highly expressed in astrocytes and mediates increases in COX-2 protein expression

**Fig. 1:**
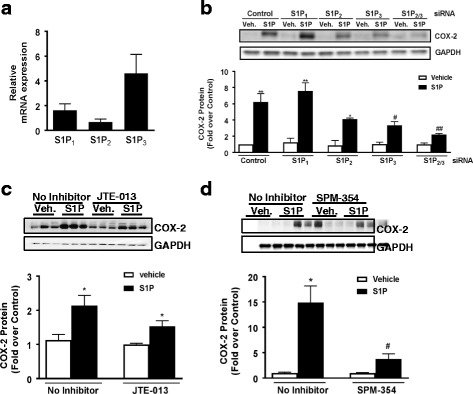
S1P_3_ is highly expressed in astrocytes and mediates COX-2 expression. In WT astrocytes, **a** S1P_1_, S1P_2_, and S1P_3_ mRNA expression was measured by absolute PCR. **b** Quantification and Western blot of COX-2 protein expression after S1P treatment (0.5 μM, 6 h) following knockdown of S1P_1_, S1P_2_, S1P_3_, and S1P_2/3_ with siRNA (2 μM). Data shown are mean ± SEM of values from three independent experiments run in triplicate. COX-2 was normalized to GAPDH and expressed relative to control siRNA vehicle treated. *p < 0.05 and **p < 0.01 between vehicle and S1P-treated groups and #*p* < 0.05 and ##*p* < 0.01 between control siRNA S1P-treated and S1P receptor siRNA S1P-treated groups. **c** Quantification and Western blot of COX-2 expression after pretreatment with the S1P_2_ antagonist JTE-013 for 30 min (1 μM) followed by S1P treatment (0.5 μM, 6 h). Data shown are mean ± SEM of values from three independent samples. COX-2 was normalized to GAPDH and expressed relative to vehicle control. *p < 0.05 between vehicle and S1P-treated groups. **d** Quantification and Western blot of COX-2 expression after pretreatment with the S1P_3_ antagonist SPM-354 for 15 min (5 μM) followed by S1P treatment (0.5 μM, 6 h). Data shown are mean ± SEM of values from three independent experiments run in triplicate. COX-2 was normalized to GAPDH and expressed relative to vehicle control. *p < 0.05 between vehicle and S1P-treated groups and #*p* < 0.05 between control S1P-treated and SPM-454/S1P-treated group

We used q-PCR to compare directly the levels of mRNA expression for S1P_1-3_ in the cultured mouse astrocytes used in the studies presented here. Relative expression levels for S1P_3_ (4.7) > S1P_1_ (1.6) > S1P_2_ (0.7) mRNA were established using absolute quantitative-PCR (Fig. 1a). To examine S1P receptor subtype involvement in induction of the inflammatory gene COX-2, all three receptor subtypes were knocked down prior to treatment with S1P. Knockdown with S1P_1_ siRNA (78% decrease in S1P_1_ mRNA) had no effect on COX-2 protein expression in cells stimulated for 6 h with S1P. Knockdown of S1P_3_ (80%) significantly attenuated *S*1P-stimulated COX-2 expression, and the combination of S1P_2_ and S1P_3_ knockdown was most effective (Fig. 1b). Knockdown of S1P_2_ (83%) diminished but did not significantly decrease S1P-induced COX-2 protein expression nor did pretreatment with the selective S1P_2_ antagonist JTE-013 (Fig. 1c). In contrast, SPM-354, a bitopic antagonist that has a significantly higher affinity for S1P_3_ than for S1P_2_ (1840-fold) or S1P_1_ (30-fold) [45], decreased S1P-induced COX-2 expression by more than 70% (Fig. 1d) further confirming the predominant role of S1P_3_ activation in COX-2 protein expression. The S1P_2_ and S1P_3_ receptor subtypes can serve redundant functions in regulation of RhoA and other downstream responses in some cell systems [36, 49] but our data indicates that S1P_3_ mRNA is most highly expressed in mouse astrocytes and plays the predominant role in mediating COX-2 protein expression in response to S1P.

### S1P_3_ is required for induction of inflammatory genes in astrocytes

**Fig. 2:**
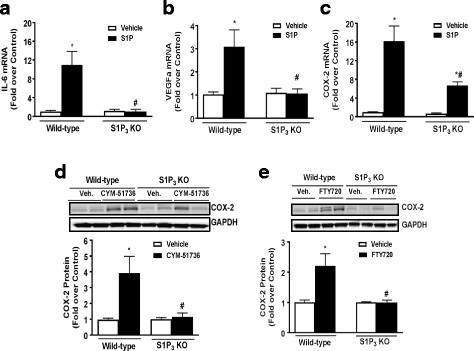
S1P_3_ is required for induction of inflammation in astrocytes. WT and S1P_3_ KO astrocytes were treated with vehicle or S1P (0.5 μM) for 1 h, and IL-6 (**a**), VEGFa (**b**), and COX-2 (**c**) mRNA expression was measured by q-PCR. IL-6, VEGFa, and COX-2 were normalized to GAPDH, and fold increase expressed relative to the WT or KO vehicle treated. Data shown are mean ± SEM of three independent experiments run in triplicate. *p < 0.01 between vehicle and S1P-treated groups and #*p* < 0.01 between WT and KO S1P-treated groups. **d** Quantification and Western blot of COX-2 expression in WT and S1P_3_ KO astrocytes treated with CYM-51736 (10 μM, 6 h). Data shown are mean ± SEM of values from three independent experiments run in triplicate. COX-2 was normalized to GAPDH and expressed relative to the WT or KO vehicle treated. **e** Quantification and Western blot of COX-2 expression in FTY720-treated (100 nM, 6 h) WT and S1P_3_ KO astrocytes. Data shown are mean ± SEM of values from three independent experiments run in triplicate. COX-2 was normalized to GAPDH and expressed relative to the WT or KO vehicle treated. *p < 0.01 between vehicle and treatment groups and #*p* < 0.01 between WT and KO treatment groups

To provide further evidence that S1P_3_ is the receptor on astrocytes that is primarily responsible for the induction of COX 2 and other inflammatory genes, astrocytes were isolated from WT and S1P_3_ KO mice. The ability of S1P to increase inflammatory gene mRNA was examined at 1 h treatment since it was demonstrated that mRNA for IL-6 and COX-2 were significantly increased by S1P at this early time [40]. Deletion of S1P_3_ fully prevented S1P-mediated increases in IL-6 (Fig. 2a) and VEGFa (Fig. 2b) mRNA. Induction of COX-2 mRNA (Fig. 2c) was markedly but not fully attenuated paralleling the changes in COX-2 protein expression shown in Fig. 1b which suggests some redundancy in S1P_2_ and S1P_3_ signaling to COX2 expression. Next, we used a recently generated S1P_3_ receptor allosteric agonist, CYM-51736, which is more specific than the previous S1P_3_ agonists [50, 51]. CYM-51736 increased COX-2 protein in WT but not in S1P_3_ KO astrocytes (Fig. 2d), consistent with its specificity and the ability of S1P_3_ activation to regulate COX-2 mRNA expression. Finally, we tested FTY720 (fingolimod), which acts as an agonist at both S1P_1_ and S1P_3_. FTY720 treatment increased COX-2 protein expression in WT but not in S1P_3_ KO astrocytes (Fig. 2e), suggesting that its agonist actions at S1P_3_ induce astrocyte inflammatory genes.

### S1P_3_ signals through Gα_12/13_ and RhoA to induce gene expression

**Fig. 3:**
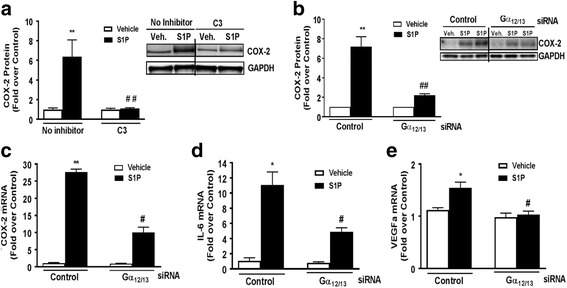
S1P signals through Gα_12/13_ and RhoA to induce COX-2 expression. **a** COX-2 protein expression was measured in WT cells pretreated with C3 exoenzyme (0.5 μg/mL) for 4 h prior to vehicle or S1P (0.5 μM) treatment for 6 h. COX-2 was normalized to GAPDH and expressed relative to the averaged ± inhibitor controls. Representative Western blot and data shown are mean ± SEM from three independent experiments run in triplicate. The *blot* represents a single gel where unnecessary lanes have been removed. **p < 0.01 between vehicle and S1P-treated groups and ##*p* < 0.01 between S1P-treated ± inhibitor groups. **b** Quantification and Western blot of COX-2 protein levels after knockdown of Gα_12/13_ with siRNA (2 μM) followed by S1P treatment (5 μM, 6 h). The blot represents a single gel where unnecessary lanes have been removed. Data shown are mean ± SEM of values from four independent experiments run in triplicate. COX-2 was normalized to GAPDH and expressed relative to the siRNA control. mRNA expression levels of COX-2 (**c**), IL-6 (**d**), and VEGFa (**e**) were measured by q-PCR following knockdown of Gα_12/13_ with siRNA (2 μM) and S1P treatment (0.5 μM, 1 h). Data shown are mean ± SEM from three independent experiments run in triplicate. COX-2, IL-6, and VEGFa were normalized to GAPDH and fold increase expressed relative to the vehicle-treated control siRNA. *p < 0.05 and **p < 0.01 between vehicle and S1P-treated groups and #*p* < 0.05 and ##*p* < 0.01 between control and Gα_12/13_ S1P-treated groups

The Gα_12/13_ proteins are the G-protein family members that most effectively couple GPCRs to RhoA exchange factors and thus to activation of RhoA. To demonstrate that S1P_3_ activates inflammatory gene expression by signaling through Gα_12/13_, we used siRNAs to achieve combined knockdown of Gα_12_ and Gα_13_ (92% decrease in Gα_12_ mRNA and 90% decrease in Gα_13_ mRNA). In addition, RhoA was functionally inhibited by pretreatment of cells with C3 exoenzyme. Both interventions significantly decreased S1P-mediated increases in COX-2 protein (Fig. 3a, b). We further demonstrated that induction of COX-2, IL-6, and VEGFa mRNAs by S1P were attenuated by knockdown of Gα_12_ and Gα_13_ (Fig. 3c–e).

**Fig. 4:**
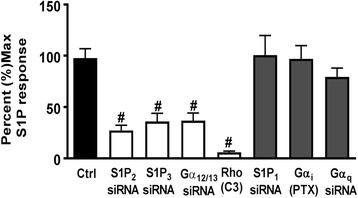
S1P_2_, S1P_3_, and Gα_12/13_ are required for Rho activation in astrocytes. WT astrocytes were transfected with an SRE.L luciferase reporter construct to assess Rho activation following knockdown with control siRNA or siRNA (2 μM) targeting S1P_1_, S1P_2_, S1P_3_, Gα_12/13_, or Gα_q_. Cells were also treated with pertussis toxin (PTX, 100 ng/mL, 24 h) to inhibit Gα_i_ or with the C3 exoenzyme (0.5 μg/mL, 4 h) to inhibit Rho. Following knockdown or pretreatment, cells were stimulated with S1P (0.5 μM, 8 h). Control siRNA treated cells stimulated with S1P were set at 100% response. Data shown are mean ± SEM from four independent experiments run in duplicate. #*p* < 0.05 between control and siRNA S1P-treated groups

The SRE.L luciferase reporter gene contains a truncated TCF-independent binding site for serum response factor (SRF) and is widely used as a readout for activated RhoA, which regulates genes through SRF and its transcriptional co-activator MRTF-A [52, 53]. S1P markedly increased SRE.L luciferase activity (16 to 50-fold, depending on the experiment). The S1P response (shown as 100% in the averaged experiments in Fig. 4) was attenuated by functional blockade of Rho signaling with C3 treatment and by knockdown of S1P_2_, S1P_3_, and Gα_12/13_, but not by knockdown of S1P_1_ or Gα_q_. Inhibition of Gα_i_ function by pretreatment with pertussis toxin (PTX) was also without effect on S1P-stimulated SRE.L activation. These data demonstrate S1P_3_ and S1P_2_ coupling to Gα_12/13_ to activate RhoA, which in turn regulates COX-2 and other inflammatory genes in astrocytes.

### S1P_3_ and Sphk1 are upregulated in response to in vitro wounding and mediate COX-2 expression

**Fig. 5:**
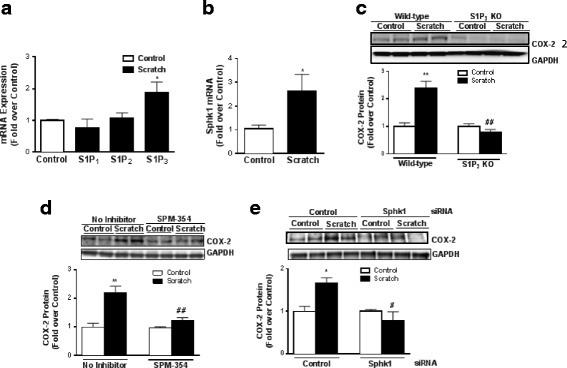
S1P_3_ and Sphk1 are upregulated in response to in vitro scratch injury and mediate COX-2 expression. In WT astrocytes, **a** S1P_1_, S1P_2_, S1P_3_ and **b** SphK1 mRNA expression were measured 1 h after scratch injury on culture plates. Data shown are mean ± SEM from three independent experiments run in triplicate. Fold increase is relative to the scratch control for each receptor subtype or Spkh1. *p < 0.05 between control and scratch-treated groups. COX-2 protein levels were measured after 8 h of scratch in **c** S1P_3_ KO astrocytes or **d** WT astrocytes pretreated with SPM-354 (5 μM, 15 min). COX-2 was normalized to GAPDH and expressed relative to the WT or KO controls or the ±inhibitor controls. Data shown are mean ± SEM from three independent experiments run in triplicate. **e** COX-2 protein levels were measured in WT astrocytes after knockdown of Sphk1 with siRNA (2 μM) or control siRNA followed by scratch for 8 h. COX-2 was normalized to GAPDH and expressed relative to the ±siRNA controls. Data shown are mean ± SEM from three independent experiments run in duplicate. * *p* < 0.05 and **p < 0.01 between control and scratch-treated groups and #*p* < 0.05 and ##*p* < 0.01 between scratch-treated groups of either WT and KO, with or without inhibitor, or siRNA

To determine whether the signaling pathway delineated above could be activated under pathophysiological conditions, we used an in vitro scratch injury model to simulate localized inflammation of cultured astrocyte [47]. Within an hour after cells were scratched, S1P_3_ (but not S1P_1_ or S1P_2_) mRNA was increased relative to control unscratched cells (Fig. 5a) as was the mRNA level for sphingosine kinase 1 (Sphk1), the enzyme that catalyzes the synthesis of S1P (Fig. 5b) [54]. Our previous studies showed that injuring astrocytes increases COX-2 expression and that the media from scratched cells contains substances that contribute to this response [40]. To determine whether this localized inflammatory response could be mediated by the actions of S1P, formed from the elevated Sphk1 and acting on astrocyte S1P_3_, we repeated the scratch injury studies using S1P_3_ KO astrocytes. The increase in COX-2 protein expression was lost, indicating that the ability of scratch to elicit this response requires S1P_3_ (Fig. 5c). We confirmed this further by comparing wild-type cells that were subject to scratch injury in the presence or absence of the S1P_3_ inhibitor SPM-354 used in Fig. 1d. Pharmacological blockade of S1P_3_, like genetic deletion of the receptor, prevented scratch-induced COX-2 expression (Fig. 5d). Finally, to test the importance of localized formation of S1P, we knocked down Sphk1 using siRNA (90% reduction in Sphk1). Scratch injury failed to increase COX-2 protein expression when Sphk1 was downregulated (Fig. 5e) implicating this enzyme, its product, and S1P_3_ activation in localized astrocyte COX-2 induction.

## Discussion

Neuroinflammation, which underlies many neurodegenerative processes including those involved in Alzheimer’s disease, Parkinson’s disease, and MS, is increasingly recognized as a hallmark of CNS pathology [19, 20, 55–58]. Astrocytes were once considered as structural elements in the brain but subsequently emerged as functionally important for neuronal guidance, maintenance of the BBB, and structural and metabolic support of neurons [15, 17, 18]. In addition, astrocytes, like microglia, are now known to contribute to neuroinflammation [14, 17, 19, 56, 59]. The lysophospholipid S1P regulates astrogliosis and inflammatory responses in the CNS; however, the role of the individual S1P receptor subtypes in these processes has not been clearly delineated [23, 30, 31, 33, 34, 60, 61].

Astrocytes contribute to neuroinflammation by upregulating proinflammatory mediators such us IL-6, MCP-1, TNF-α, iNOS, and COX-2 [15, 17, 40, 62]. Induction of COX-2 in astrocytes increases generation of reactive oxygen species (ROS), as well as formation of prostanoids that play a prominent role in inflammation, and thus further contribute to neuronal cell death and demyelination in diseases such as MS [20, 56]. Moreover, astrocytes produce VEGF which plays a role in the breakdown of the blood-brain barrier, a step critical to the entry of pathogenic lymphocytes into the brain [63–67]. Our data demonstrate that an important mechanism for induction of inflammatory cytokines and cytotoxic mediators such as IL-6, COX-2, and VEGFa in astrocytes is through their exposure to S1P and activation of S1P_3_.

Both S1P_1_ and S1P_3_ are expressed on astrocytes [8, 12] and are upregulated on reactive astrocytes that contribute to inflammation associated with CNS disease [32, 33, 35, 61, 68]. In response to inflammatory stimuli or in CNS pathologies, Sphk1, an enzyme that generates S1P, is also increased in astroglial cells [23, 34, 35, 69, 70]. Our findings using siRNA and S1P_3_ KO astrocytes demonstrate mechanistically that agonist binding to S1P_3_ signals to inflammatory responses through S1P_3_ coupling to Gα_12/13_ and activation of RhoA. We also show here, using an astrocyte scratch injury assay, that S1P_3_ and Sphk1 expression are increased by simulated inflammation and demonstrate by their knockout and downregulation, respectively, that they are involved in an autocrine signaling loop to increase COX-2 expression. While S1P_2_ could also signal through Gα_12/13_ and RhoA to contribute to COX-2 expression ([37, 71–73] and Fig. 4) and appears to serve this role when S1P_3_ is downregulated (Fig. 1b), the relatively low expression of this receptor subtype and its lack of upregulation in response to wounding suggests limited involvement in astrocyte inflammatory responses (Fig. 1c). Thus, it appears that S1P_3_, and its autocrine activation by S1P generated through Sphk1, are poised to mediate astrocytic inflammatory responses that could contribute to the progression of CNS neuropathology.

S1P signaling in the CNS has important pathophysiological consequences [21, 28–30, 33–35, 40, 61, 74]. Much research has focused on S1P_1_ as the primary target for the MS drug FTY720 (fingolimod). While a well-recognized effect of fingolimod is to functionally antagonize S1P_1_ receptors on lymphocytes and thereby prevent their egress into the blood and access to the brain, S1P_1_ localized to astrocytes contributes significantly to the effects of this drug in an experimental model of MS [75]. The basis for also considering S1P_3_ signaling in MS is that this receptor subtype is upregulated in astrocytes during MS and in EAE and that it is a target for fingolimod [33, 61]. Notably, fingolimod causes transient bradycardia that appears, at least in the mouse, to be due to its agonist actions on S1P_3_ [24, 25, 76–78]. While it is clear that fingolimod downregulates S1P_1_, and thus acts as a functional antagonist, its ability to similarly downregulate and thus act as a functional antagonist of S1P_3_ is controversial [61, 77, 79, 80]*.* A recent study demonstrated that continuous treatment with FTY20, initiated at the onset of disease in an EAE model, reduced S1P_3_ expression at day 22 [61]. While this indicates that S1P_3_ is downregulated by FTY720 treatment, this could reflect reversal of the disease process/inflammation (and its accompanying increases in S1P_3_ gene expression) rather than downregulation at the receptor level. Our data with FTY720 (like that examining bradycardia) demonstrate that FTY720 acts as an agonist, eliciting COX-2 induction, over a period of at least 6 h. Our data further establish that it is S1P_3_-mediated RhoA signaling, not effects of S1P_1_ and Gα_i_, that lead to maladaptive astrocyte inflammation. Thus, agonism at astrocyte S1P_3_ by fingolimod or other drugs could contribute to neuroinflammation and worsen disease progression, particularly when S1P_3_ are upregulated and S1P availability increased through activation of sphingosine kinase. Further studies using S1P_1/3_ double knockout mice are ongoing and should indicate whether blocking S1P_3_, in addition to S1P_1_, would have additional therapeutic benefit.

The importance of S1P_3_ and RhoA signaling in CNS disease could be logically extended to consideration of any of the myriad GPCRs found on astrocytes [32] that couple to RhoA signaling. We and others have shown that PAR1, the receptor for thrombin, couples through RhoA to mediate proliferation and inflammatory responses in astrocytes [39, 40]. Thrombin is also increased in the injured brain [81, 82], and an antagonist of protease activated receptor 1 (PAR1) reduces clinical symptoms in EAE mice [83]. Thus, the evidence that S1P_3_ and other GPCRs that stimulate RhoA can contribute to sustained inflammatory responses suggests this pathway as a critical target for blocking neuroinflammation in MS and other CNS diseases.

## Conclusions

Our findings demonstrate that S1P_3_ and Sphk1 are mediators of inflammatory signaling and are upregulated in astrocytes in response to injury. S1P_3_ couples to Gα_12/13_ and activated RhoA to induce COX-2, IL-6, and VEGFa mRNA as well as COX-2 protein expression in astrocytes. The data suggest that blocking S1P_3_, as well as the clinically relevant S1P_1_, could have therapeutic benefit for limiting CNS inflammatory disease progression.

## Abbreviations

BBB: Blood-brain barrier
CNS: Central nervous system
COX-2: Cyclooxygenase 2
EAE: Experimental Autoimmune Encephalomyelitis
GFAP: Glial fibrillary acidic protein
GPCR: G protein-coupled receptor
IL-6: Interleukin 6
KO: Knockout
MS: Multiple Sclerosis
NF-κB: Nuclear factor kappa B
PAR1: Protease activated receptor 1
PTX: pertussis toxin
q-PCR: Quantitative-PCR
S1P: Sphingosine 1-phosphate, S1P_1_, Sphingosine 1-phosphate receptor 1
S1P_2_: Sphingosine 1-phosphate receptor 2
S1P_3_: Sphingosine 1-phosphate receptor 3
S1P_4_: Sphingosine 1-phosphate receptor 4
S1P_5_: Sphingosine 1-phosphate receptor 5
Sphk1: Sphingosine kinase-1
VEGFa: Vascular Endothelial Growth Factor A
WT: Wild-type
